# Functionality and Mechanical Performance of Miniaturized Non-Assembly Pin-Joints Fabricated in Ti6Al4V by Laser Powder Bed Fusion

**DOI:** 10.3390/ma16216992

**Published:** 2023-10-31

**Authors:** Florian Gutmann, Klaus Hoschke, Georg Ganzenmüller, Stefan Hiermaier

**Affiliations:** 1 Department of Sustainable Systems Engineering—INATECH, Albert-Ludwigs-University Freiburg, Emmy-Noether-Straße 2, 79110 Freiburg, Germany; 2 Fraunhofer Institute for High-Speed Dynamics (EMI), Ernst-Zermelo-Str. 4, 79104 Freiburg, Germany; klaus.hoschke@emi.fraunhofer.de

**Keywords:** additive manufacturing, non-assembly joint, Ti6Al4V, LPBF, miniaturization

## Abstract

In this work, additively manufactured pin-joint specimens are analyzed for their mechanical performance and functionality. The functionality of a pin-joint is its ability to freely rotate. The specimens were produced using laser powder bed fusion technology with the titanium alloy Ti6Al4V. The pin-joints were manufactured using previously optimized process parameters to successfully print miniaturized joints with an angle to the build plate. The focus of this work lies in the influence of joint clearance, and therefore all specimens were manufactured with a variety of clearance values, from 0 µm up to 150 µm, in 10 µm steps. The functionality and performance were analyzed using torsion testing and tensile testing. Furthermore, a metallographic section was conducted to visually inspect the clearances of the additively manufactured pin-joints with different joint clearance values. The results of the torsion and tensile tests complement each other and emphasize a correlation between the joint clearance and the maximal particle size of the powder utilized for manufacturing and the mechanical behavior and functionality of the pin-joints. Non-assembly multibody pin-joints with good functionality were obtained reliably using a joint clearance of 90 µm or higher. Our findings show how and with which properties miniaturized pin-joints that can be integrated into lattice structures can be successfully manufactured on standard laser powder bed fusion machines. The results also indicate the potential and limitations of further miniaturization.

## 1. Introduction

The ongoing developments and innovations in additive manufacturing (AM) establish a new prototyping technology in the industry. This is because AM is able to fabricate components by adding material step by step instead of removing or deforming the material. Thus, AM can realize more complex geometries that were previously impossible or too expensive to manufacture traditionally. In particular, laser powder bed fusion (LPBF) is an AM process that allows the manufacturing of complex metallic structures. Non-assembly mechanisms [1,2,3,4], lattice structures like bone scaffolds [5,6], and mechanical metamaterials are examples of such metallic complex structures. Mechanical metamaterials exhibit unusual macroscopic mechanical behavior that is governed by a specific underlying complex lattice structure also known as unit cell geometry, e.g., negative Poisson’s ratio [7], twisting under pressure loading [8], negative stiffness [9], and exceptional elastic deformation [10]. Thereby, the complexity of the unit cell geometry and the density of unit cells relates to the realization of novel material behaviors that classical materials cannot provide. Therefore, non-assemblies that allow the realization of even more complex unit cells on a small scale are of particular interest.

In this article, miniaturized non-assembly pin-joints are manufactured on a standard industrial LPBF machine, and their mechanical performance is analyzed. Non-assemblies are mechanisms that require no additional assembly step after manufacturing and are fully functional mechanisms. Particularly challenging are multibody non-assembly mechanisms consisting of separate parts [3] and actual moving joints, which are investigated in this work. Thereby, the joint clearance is of particular interest. On the one hand, a large clearance leads to vibrations and instabilities of non-assemblies and their functional structures [4,11], and on the other hand, a small clearance creates a more accurate mechanism but can cause a potential fusion of the separate parts. By optimizing the design of the joint using a combination of concave and convex shapes, support structures are unnecessary within the joint clearance [3,12,13]. To obtain a higher-quality joint while manufacturing joints with an angle to the build plate, a reduction of maximal particle size and an optimization of process parameters have been recommended in the work of Boschetto et al. [13] and Su et al. [11]. Furthermore, a warpage in the build direction that could potentially lead to a fusing of the joint has been observed.

It is crucial to avoid geometrical deviations of the additive manufactured part to the computer-aided design (CAD) model, as observed in other studies [14,15,16], to ensure the functionality of non-assembly mechanisms such as a joint with a small clearance. The smaller the structure is, the bigger the impact of the surface roughness on the geometrical deviation is. Calignano et al. [17] pointed out that the surface roughness for filigree structures is primarily defined by partially fused particles sticking to the molten metal. The geometrical accuracy of additive manufactured parts is further influenced by thermal stress and process parameters. Thus, optimized process parameters are highly recommended to fabricate miniaturized pin-joints.

The main focus of this study is to investigate the mechanical performance of miniaturized non-assembly pin-joints in relation to their nominal joint clearance, from 0 µm (a rigid joint) up to 150 µm, to showcase how and with which properties pin-joints can be integrated in lattice structures. This investigation is absolutely necessary to enable the validation and goals of certain pantographic metamaterial studies [18,19] that require a perfect pivot, meaning real pin-joints in a non-assembly that allow free rotation. Therefore, the pin-joints of this work were printed at a 45° angle to the build plate to emulate their possible integration into a lattice structure. For this investigation, torsion specimens, as suggested by Boschetto et al. [13], were manufactured and analyzed to assess the functionality of the joint, to allow the mechanical freedom of rotation. Tensile specimens were also designed and analyzed to obtain further information on the joint’s mechanical performance. Furthermore, a specimen was manufactured on which a metallographic section was conducted to reveal the manufactured clearance in the plane through the pin-joints where the biggest deviations compared to their CAD model were to be expected. All specimens were manufactured on a standard LPBF machine using optimized process parameters that, in a previous study by Gutmann et al. [20], featured the successful manufacturing of miniaturized pin-joints with a constant joint clearance of approximately twice the maximal particle size (120 µm).

## 2. Materials and Methods

### 2.1. Design of the Pin-Joint and the Testing Specimens

**Figure 1:**
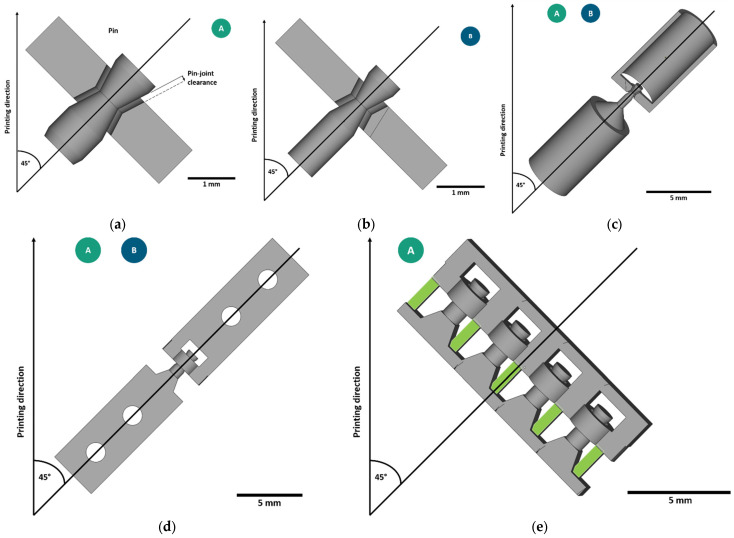
CAD illustration of the miniaturized pin-joints and their deduced testing specimens: (**a**) pin-joint A (pin Ø 0.56–1 mm); (**b**) pin-joint B (pin Ø 0.32–0.66 mm), a further miniaturization of pin-joint A; (**c**) torsion specimen illustrated with pin-joint B; (**d**) tensile specimen illustrated with pin-joint A; and (**e**) the metallographic section specimen. The green-marked surfaces show the plane where the metallographic analysis (see Section 2.4) is performed.

The pin-joints had edged concave-shaped holes and edged convex-shaped pins. The edging of the geometrical shape simplified the model and reduced the number of different angles within each joint for downscaling purposes. Figure 1 depicts the two miniaturized pin-joints (A and B) and their deduced testing specimens (c, d and e). The bigger variant of the miniaturized pin-joints, pin-joint A (a), had the same geometry as the pin-joints integrated in a pantographic metamaterial with a constant clearance of 120 µm in a previous study by Gutmann et al. [20]. Pin-joint B (b) was a further realized miniaturization of pin-joint A by approximately 33%. The diameter of the pin was reduced from 0.56–1 mm to 0.32–0.66 mm, and accordingly, the beam’s thickness was equal to the maximal value of the pin’s diameter. Thereby, the most acute angle of the overhanging surfaces of the non-assembly pin-joints was 27.5°.

In this study, the joint clearance of all specimens was varied in 10 µm steps to analyze the impact on the manufacturability and mechanical performance of both pin-joint variants. Thereby, the geometry of the pin was kept constant, and the joint clearance was subtracted from the surrounding material. The joint clearance of the torsion specimens (c) was varied from 0 to 130 µm; for the tensile specimens (d) and metallographic section specimens (e), it was varied from 0 to 150 µm. The metallographic section specimens were only manufactured with pin-joint A and four different joint clearance values in each specimen.

### 2.2. Additive Manufacturing

An EOS M 100 LPBF machine (EOS GmbH, Krailling, Germany) was used for the production of the specimens. The machine was equipped with a 200 W laser unit (YLR-series, CW-laser, wavelength 1070 nm) that had a focus diameter of 40 µm. The titanium alloy powder was applied in a layer thickness of 20 µm. It consisted of a mixture of sieved (63 µm mesh) and unused Ti6Al4V powder, and the mix was further investigated with a particle analysis. To avoid oxidation, an argon-based inert gas atmosphere of O_2_ < 0.1% was applied.

For the testing specimen, an optimized process parameter set was used for manufacturing. The study by Gutmann et al. [20], utilized on the same machine setup and using powder from the same supplier with identical powder specifications, showed that this parameter set allows the realization of miniaturized pin-joints. Thereby, small melt track sizes were realized, and a high density of the material was achieved while allowing the geometrical accuracy that is a necessity for a functional pin-joint. The process parameter set was split into skin and core parameters and is summarized in Table 1.

After manufacturing, all specimens were sandblasted to remove adherent powder particles from the outer surface. No heat treatment was applied to the specimens or any other surface treatment before conducting the following examination.

### 2.3. Mechanical Analysis

The torsion and tensile specimens were mechanically tested to determine the performance of a single pin-joint under load and to analyze which nominal joint clearances allow the manufacturability of a functional joint.

#### 2.3.1. Torsion Testing

**Figure 2:**
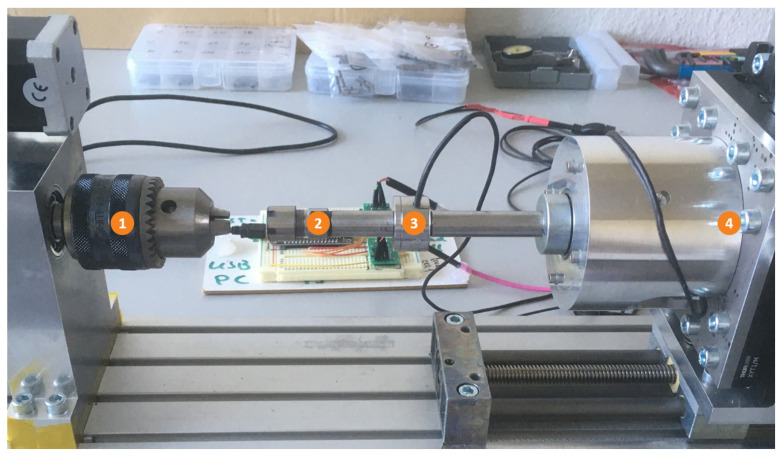
Torsion testing setup adapted from [21]. The fixed drive (1) was original; a straight milling extension (2) allowed mounting of specimens without high force that were monitored by the pressure sensor (3); the torque sensor (4) was modified to allow accurate measurement for torque up to ±1Nm.

The torsion analysis was conducted on an adapted testing device adapted from Trippel et al. [21] that is suitable to measure torque values relevant for small rotational joints and therefore suitable to test the miniaturized pin-joints of this work. The adapted torsion setup is illustrated in Figure 2. The fixed drive (1) of the device was original. The following adaptations were changes made compared to the published device. The drill chuck was exchanged with a (2) straight milling extension ER11 with dimensions: D:12, L:140 (Gedema^®^ GmbH, Hagen, Germany), and a (3) pressure sensor KM26z 500 N (ME-Meßsysteme GmbH, Henningsdorf, Germany) was added to the extension rod. These changes allowed the measurement of the force applied along the pin-joint axis during torsion and while mounting a specimen into the setup. This way, the axial loading could be adjusted while fastening the specimens to the device. Strong forces that might damage the pin-joint could be avoided, and the axial force could be zeroed before the torque measurement was started. Furthermore, the (4) torque sensor was exchanged with a TD70 ±1 Nm/VA torque sensor (ME-Meßsysteme GmbH, Henningsdorf, Germany).

The specimens were torqued on this adapted setup up to four full turns (4 × 360°) or until failure. The testing speed for this investigation was set to 4.5°/s. The torque and axial force were measured simultaneously.

#### 2.3.2. Tensile Testing

**Figure 3:**
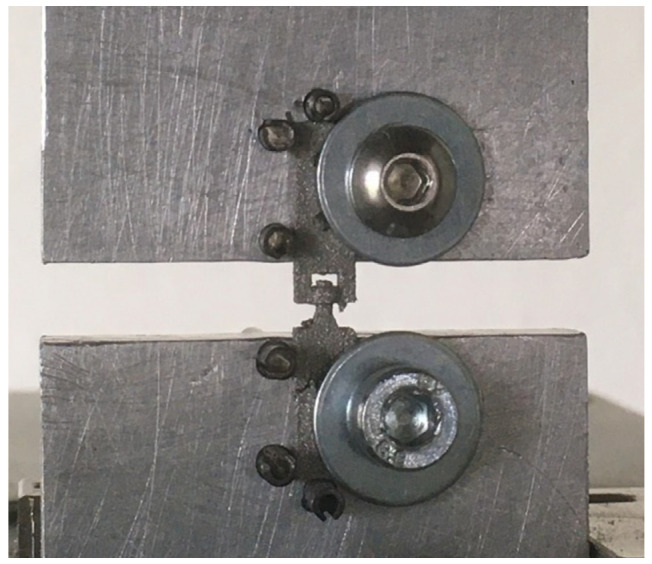
Mounted clamping platform for the tensile testing setup depicted with a pin-joint A tensile specimen.

For the tensile testing, the specimens were pulled using a Zwick RetroLine testing machine (ZwickRoell AG, Ulm, Germany) in displacement control mode. The tests were conducted until failure with a velocity of 2 mm/min. Because of their size, the specimens were mounted on specially manufactured platforms, as shown in Figure 3, which were clamped with screw grips. This allowed easier access to insert and remove the specimen. Furthermore, this prevented strong forces from tightening the screw grips, which may have damaged and destroyed the miniaturized pin-joints.

### 2.4. Metallographic Section and Microscopic Investigation

The specimens were prepared by standard metallographic means using a polishing machine Tegrapol 35 (Struers GmbH, Willich, Germany) with a rotational speed of 180 rpm. The specimens were manually polished in steps until the median plane was approached, which is indicated by the uniform removal of the green-marked pillars in Figure 1e.

The prepared specimens were analyzed using light microscopy regarding the aspects of shape and the actual size of the manufactured clearance of the pin-joints in the median plane. The median plane was examined since it showcased the surfaces with the most acute angles of the pin-joint structure with regard to the build plate. At these acute angles, the impact of the staircase effect and poorer quality of overhanging surfaces, commonly observed in LPBF [17,22,23], was the largest. This optical investigation was conducted on a ZEISS Axio Imager.Z2m (Carl Zeiss AG, Oberkochen, Germany). The images published in this article were generated by taking images with a 5× magnification, which were then combined into a single tiff file using the program ZEISS ZEN core.2.5. For the powder analysis, a CAMSIZER^®^ X2 (Micotrac MRB, Haan, Germany) was used to analyze the manufacturing Ti6Al4V powder (EOS Titanium Ti6Al4V, EOS GmbH, Krailling, Germany). The setup offers a particle size measurement range from 0.8 µm to 8 mm. A volume distribution was computed using the data of a dynamic image analysis (ISO 13322-2 [24]) which was conducted with a dispersion pressure of 30 kPa.

## 3. Results and Discussion

### 3.1. Powder Analysis

**Figure 4:**
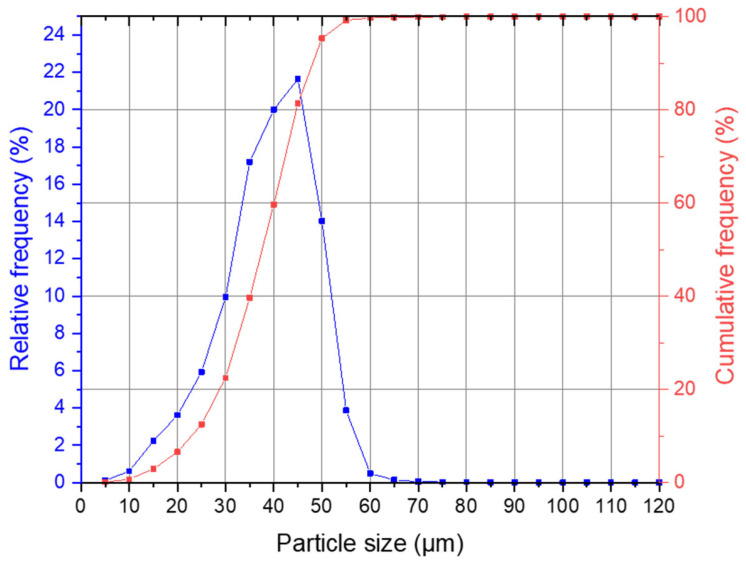
Ti6Al4V powder particle size distribution.

The particle size analysis of the Ti6Al4V powder showed a cumulative particle size distribution of D10: 23.27 µm, D50: 37.64 µm, and D90: 47.65 µm; the full measurement is illustrated in Figure 4. The particle shape analysis calculated a width/length ratio (aspect ratio) b/l3 of 0.869 and a specific surface area S_v_ of 180.06 mm. From the area-to-perimeter ratio, a mean sphericity SPHT3 of 0.872 was calculated; for a perfect sphere, this value was equal to 1.

### 3.2. Additive Manufacturing and Microscopic Investigation

**Figure 5:**
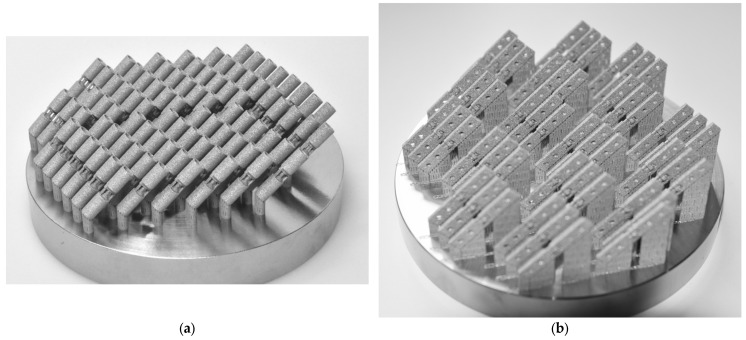
As-built manufactured specimens with support structures on Ø 100 mm build plates: (**a**) torsion specimens; (**b**) tensile specimens.

The manufactured torsion and tensile specimens in as-built condition are shown in Figure 5. No particular errors of the manufactured specimens were observed. They were removed with a small side cutting nipper from the build plate undamaged, the two small support structures right next to the actual joint stabilized it and prevented damage prior to their testing. These joint stabilizing support structures were only removed after the specific specimen was mounted on their associated testing device successfully.

**Figure 6:**
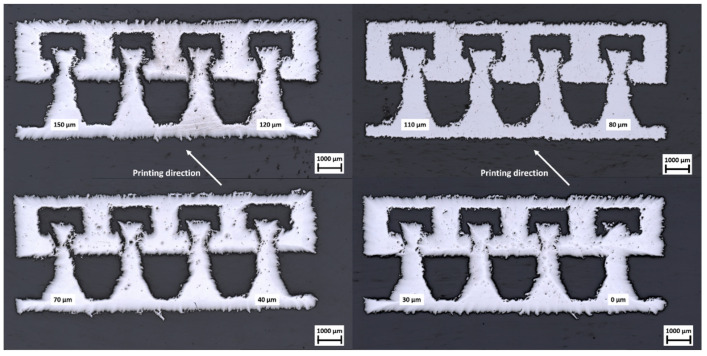
Imaging of the metallographic sections with decreasing nominal pin-joint clearance from the top left (150 µm) to bottom right (0 µm).

The metallographic sections are shown in Figure 6. Edge rounding was observed on three images of the sections, which was due to multiple grinding steps to reach the median plane (the green-marked plane in Figure 1e). In this plane, the deviations of overhanging surfaces from the CAD model were the largest, and the difference in joint clearance in regard to the angle of the surfaces to the printing direction became evident. The more acute the angle of a surface to the build plate (orthogonal to the building direction) was, the bigger the deviations were. Overall, the joint clearance became more defined with increasing nominal clearance size, but an overlapping of the pin with the surrounding material could be observed even in the 150 µm joint clearance specimen. Though the amount of overlapping and the area of clear material fusion increased with decreasing joint clearance up to a rigid body for the 0 µm joint clearance variant, the joint clearances for 10 µm to 30 µm looked more like an accumulation of pores along the nominal clearance compared to higher clearance values where the pin could be recognized as a separate structure. From this presented view alone, it was difficult to judge which pin-joint would function as an actual joint, so mechanical investigation was necessary. Given these images and the particle size distribution of the powder used for manufacturing, the authors expected a functional pin-joint with at least a joint clearance of more than 40 µm and potential forces needed to break loose local fusions or overlapping of material. Furthermore, a functional pin-joint without needing local forces to break the pin loose was expected, starting with a joint clearance of 70 µm, which is bigger than the size of the majority of particles measured.

### 3.3. Mechanical Analysis

#### 3.3.1. Torsion Analysis

**Figure 7:**
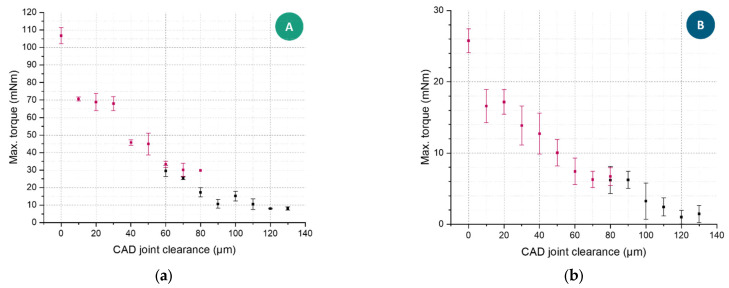
The maximal torque of the pin-joint A and B torque specimens was averaged over a set of three experiments (six if divergent results were observed), and the error bars denote the standard deviation. The red measurement values represent specimens that broke during testing, and the black values represent intact specimens after testing. (**a**) Pin-joint A torque measurements with 6 specimens for the joint clearance of 60 µm (4 broken, 2 intact), 70 µm (4 broken, 2 intact), and 80 µm (1 broken, 5 intact); (**b**) pin-joint B torque measurements with 6 specimens for the joint clearance of 80 µm (4 broken, 2 intact).

To analyze the functionality of the joints, torsion tests were performed on the pin-joints with varying nominal joint clearances. If a sample group of three specimens with the same clearance showed differing functionality (functional and breaking) during torsion, the size of the sample group was doubled. In Figure 7, the maximal torque in relation to the two pin-joints and their joint clearance is illustrated. Thereby, functional joints are marked black, and broken joints are marked red. It was observed that a torque of approximately 29 mNm for pin-joint A and approximately 6 mNm for pin-joint B could be considered a threshold between the pin breaking due to torsion and the pin being able to break free of local fusions without breaking. To put these values into perspective, the contact between the inner and outer cylindrical surfaces of conventional pin-joints by assembly without any load is usually assumed to be frictionless, and therefore any torque can be considered an imperfection.

The rigid joint (0 µm clearance) sustained distinctly higher torsions than any specimen with an actual clearance because the effective diameter of the specimen is nearly twice the smallest diameter of the pin structure. Starting from 10 µm clearance, a roughly linear drop of applied torque could be observed until it seemed to reach a minimum plateau (fully functioning joints) and the specimens did not break anymore. It can be concluded that the lower the measured torque was, the lower the number of overlapping structures and fusions were in the clearance area. The authors assumed that the remaining torque observed in fully functional joints was due to the friction of touching surfaces within the joint clearance, and it amounted to approximately 10 mNm for pin-joint A and approximately 2 mNm for pin-joint B.

In direct comparison, pin-joints A and B behaved very similar during torsion, though the torque was roughly five-times lower for the further miniaturized pin-joint. The lowest joint clearance value that did not lead to any broken specimens was 90 µm in both cases, although pin-joints A showed the ability to sustain their functionality in the range of 60–80 µm more often. This function sustainment difference is explained by the pin’s stiffness increase in the bigger pin cross-section area compared to the critical surface area increase of the overhanging structure that is mainly affected by the surfaces in the median plane, where the metallographic section (Figure 6) was conducted.

**Figure 8:**
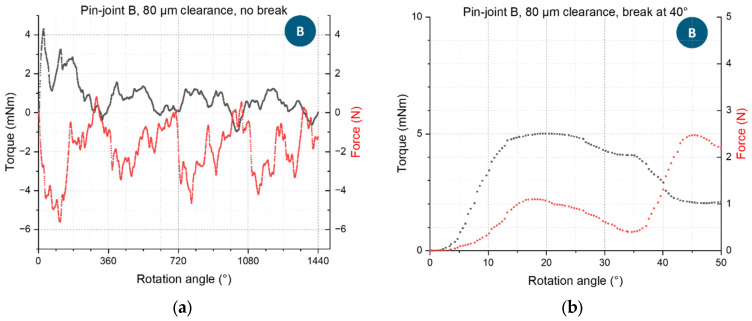
Selected torque-rotation-angle curves of pin-joint B specimens with an 80 µm joint clearance: (**a**) full measurement of torque and axial force of 4 full rotations without the specimen breaking; (**b**) measurement of torque and axial force of a specimen with the same nominal joint clearance that breaks at approximately 40°. Thereby, the force was measured simultaneously to the torque along the axis of the pin and therefore along the milling extension of the setup, as featured in Figure 2.

Figure 8 shows for pin-joints B and an 80 µm clearance the cyclic torsion behavior and a measurement of a specimen with a pin that broke. The necessary torque for continuous rotation reduced with each full turn due to breaking free of local fusions and detrition of touching surfaces. Furthermore, the necessary torque is dependent on the rotational angle and shows cyclically repeating local maxima and minima. This is explained by the not perfectly round pins and holes of the pin-joint structure. Accordingly, the measured axial force showed similar cyclic behavior with regard to the torque. Furthermore, the negative axial force values indicate that surface irregularities in the pin-joint during the rotation slightly pushed the two bodies of the specimen together. Positive axial force values indicate a potential torsion and elongation of the pin or other surfaces interactions that the two bodies of the specimen apart. Any axial force affects the torsion behavior of the specimen and should be recorded. The axial force values are rather low compared to the sustainable forced measured by the following tensile testing. Therefore, the axial force can be considered to not be the leading cause of the pins breaking during torsion. Figure 8b shows torque and axial force curves for the case of a specimen that breaks under torsion. It can be observed that the torque rapidly fell while the force pushing the pin-joint apart increased due to the elongation of the torqued pin and possible irregularities on the fractured surfaces forcibly being pushed against each other.

#### 3.3.2. Tensile Analysis

**Figure 9:**
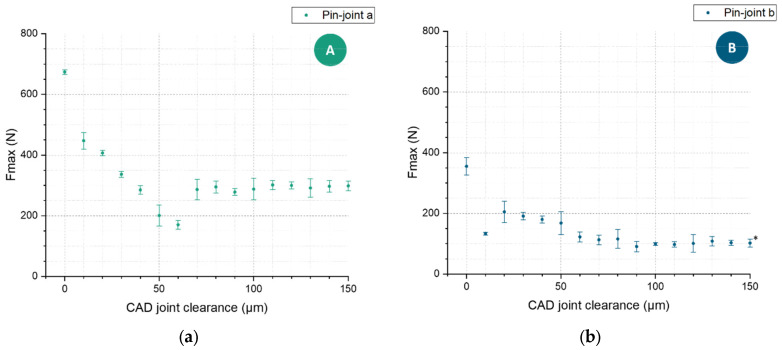
The maximal axial force is averaged over a set of three individual experiments. The error bars denote the standard deviation. (**a**) shows the results of the tensile specimens with pin-joint A, and (**b**) shows the results of the pin-joint B variants, respectively. In ()* two of the three 150 µm specimens, the multibody separated without the pins breaking.

To complement and verify the previous results, tensile tests were performed on single pin-joint specimens with up to a 150 µm joint clearance. The ultimate force that the pin-joints could sustain in relation to their joint clearance is illustrated in Figure 9. Considering the given cross section for the rigid joint and the functional pin-joint of each specimen, the rigid joint in comparison to the pin-joint was expected to sustain a force 3.19 times higher for pin-joint A and 4.27 times higher for pin-joint B. The presented tensile results for the rigid joints and the pin-joints that can be considered functional through the torsion test roughly fit these multipliers and therefore show a clear correlation of ultimate force to the effective cross section area of the pin, which stays the same for functional pin-joints since the joint clearance is subtracted from the surrounding material. Nevertheless, the measurements for the pin-joints with a clearance of 10 to 60 µm showcased a differing mechanical performance. They showed a decreasing sustaining maximal load with increasing joint clearance, which can even be below the load capacity of the functional pin-joints, for example, pin-joints A with 50 µm and 60 µm joint clearance and pin-joint B with 10 µm joint clearance. An explanation for this could be that in this range from 10 to 60 µm, which is smaller than the biggest measured particle size of the powder used for manufacturing, there is an amount of fused material in the joint clearance that correlates with the particle size distribution and the size of the joint clearance. This correlation is backed by the observation of adhering powder particles on LPBF surfaces [17,25], especially at overhanging surfaces. Furthermore, specimens with exactly the same fabrication as the pin-joints show adhering powder particles in scanning electron microscopy and computer tomography images [20,26]. Therefore, an adhering powder particle can potentially bridge the gap of the joint clearance of 10 to 60 µm leading to local fusion. The smaller the joint clearance is, the more fused the pin is with the surrounding material; the metallographic section (Figure 6) also supports this claim. This partial fusion leads to partial reinforcement compared to functional joints due to increased cross section, but at the same time, a weakening of the material behavior is observed, which is similar to the effect of an increasing number or volume of pores in the material [27]. This weakening explains the relative strong loading drop from 0 to 10 µm joint clearance and the observed underperformance for specific joint clearances, where it is likely that the resulting porous structure initiated an early crack in the pin. These load capacity changes fell a bit short for pin-joint B, which can be explained by the size effect observed by Yin et al. [26].

This tensile strength investigation also shows an upper limit for the joint clearance. The clearance of 150 µm for pin-joint B led to two-thirds of the specimens separating during loading without the pins breaking; the pins slipped with some abrasion through the hole of the surrounding material. To avoid this, a diameter difference of the minimal hole diameter, affected by the applied joint clearance and the geometrical angles of the joint structure, to the maximal pin diameter is necessary. In this context, the angle in the overhanging part of the pin-joint also defines the necessary height of the whole pin-joint geometry to guarantee the necessary diameter difference. Thus, the smaller the angle of the overhanging surface is to the build plate, the smaller, in total size, the pin-joint can be constructed. The quality and geometrical accuracy of overhanging surfaces, however, also depend on the combination of this angle and the manufacturing process parameters and remain a challenge without the introduction of support structures. Therefore, a limiting factor for the downscaling of pin-joints that can be integrated in lattice structures is how accurately surfaces with an acute angle to the build plate can be manufactured.

**Figure 10:**
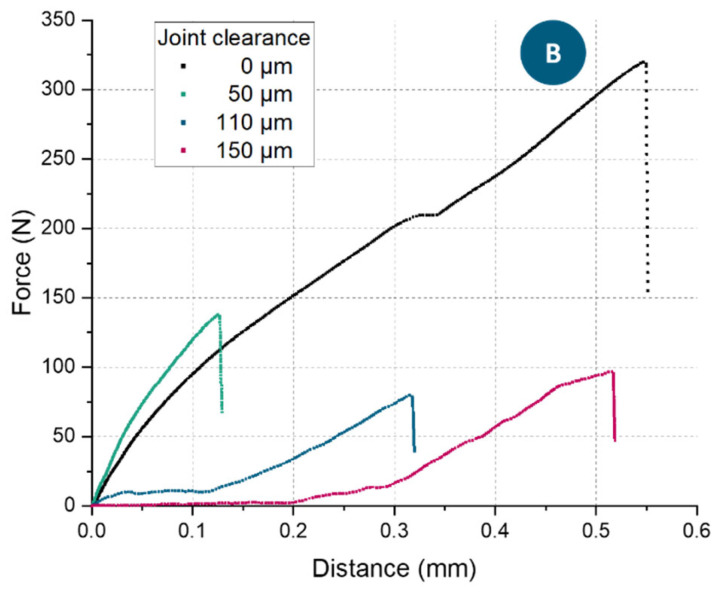
Selected force-displacement curves of pin-joint B specimen’s tensile testing. Without a nominal clearance (black curve, rigid joint), the effective diameter of the pin was 0.66 mm. With an actual joint clearance, the smallest diameter of the pin was reduced to 0.32 mm, and the maximal force the pin-joint could sustain falls accordingly (green curve). With a nominal joint clearance of 110 µm (blue curve), a sliding of the pin could be observed until the pin came in contact with the surrounding material. A further increase in joint clearance (red curve) led to a longer sliding distance.

In addition to the measured data of the maximal loading capacity of the pin-joints, selected force-displacement curves of these tensile tests with pin-joint B are illustrated in Figure 10. The curve of the rigid joint, compared to the other curves, displayed the expected higher loading capacity until breakage of a bigger tensile sample. By contrast, the curve of the 50 µm clearance specimen exhibited drastically different features. The maximal force was reduced by more than half compared to the rigid joint, and the travel distance quartered its value. This can be explained by the manufacturing size effect; the correlation of pore volume to surface-to-volume ratio of microbeams, described by Yin et al. [26]; and the mechanical change of a tensile test of a single body to that of a multibody system, introducing friction, abrasion and potential transverse forces due to rough surfaces interacting. Thereby, the rough surface can lead to the multibody interlocking at a slight angle to the pin’s axis, introducing potential transverse forces. It is also possible that the rough surfaces temporarily interlock until some abrasion on either of the bodies happens. With increasing joint clearance, starting at 90 µm, a plateau in the force measurement was observed. This plateau in the force is explained by the size of the joint clearance and represents the distance needed for the two bodies to become fully in contact. Therefore, the appearance of this plateau in the force-displacement curve can be considered an affirmation of a functional pin-joint.

## 4. Conclusions

In this work, the mechanical performance of non-assembly pin-joints manufactured at a 45° angle with respect to the build plate by LPBF was investigated. In particular, specimens were manufactured for tensile and torsion strength as well as metallographic section analysis with optimized process parameters. By evaluating the necessary torque for rotation, the functionality of the pin-joints was verified for two different steps of miniaturization in relation to differing nominal joint clearance values.

No functional pin-joints could be obtained with a joint clearance from 0 µm to 50 µm. It was shown that pin-joints with a joint clearance of 60 µm, roughly a little smaller than the size of the maximal powder particle used for manufacturing, can be functional joints, but not at a reliable rate, as four out of six samples failed. All specimens with a clearance of 90 µm or higher withstood the torsion testing without breaking. Therefore, the authors suggest, if optimized process parameters together with a suitable base material are used, a minimum joint clearance of 1.5–2 times the maximal particle size to manufacture functional miniaturized pin-joints, e.g., for integration into a lattice structure.

Considering the mechanical performance, the manufactured pin-joints can be divided into four categories. First, the rigid joint, which behaves as a single body. Second, the porous joint, where the pin-joint is primarily affected by the porous structure manufactured in the joint clearance. Third, the partly fused joint, where the pin-joint has to initially and successfully break partly fused areas or particles to obtain its function. Lastly, there is the true multibody pin-joint, which has negligible rotational torque requirements.

With the findings of this work, the authors conclude that further miniaturization of pin-joints that can be integrated in lattice structures is limited by the geometrically accurate manufacturability of overhanging structures. To be more specific, the angle of overhanging surfaces to the build plate correlates to the minimum achievable total size of the pin-joint structure due to the necessary geometrical diameter offset requirements of the pin-joints’ pin and hole, whose size values depend on the same angle. Furthermore, any geometrical deviations increase the necessary size of the nominal joint clearance, resulting in an increased total pin-joint size since the diameters of the pin and hole need to be adapted accordingly. Therefore, the more accurate overhanging structures with a smaller angle to the built plate can be manufactured, the smaller the pin-joint can be miniaturized.

Further investigations can be performed on the geometrical compensation of the CAD model, up to the maximal particle size used, to improve the circularity of the manufactured pin and hole. Furthermore the feasibility of a reduction in particle size of the powder used for manufacturing on standard LPBF machines can be investigated, as well as if the findings of this work, in regard to the correlation of functionality to the multiplication factor of the particle size to the joint clearance, can be reproduced.

## Figures and Tables

**Table 1:** Process parameter set used for the manufacturing of the testing specimen with miniaturized pin-joints.

Parameter Set	Laser Track	Hatch Distance (µm)	Laser Power (W)	Scanning Speed (mm/s)
Core *	Stripes	40	70	2000
Skin	Contour (2×)	8	50	2000
	Stripes	30	50	2000

* No contour parameter for the core parameter set.

## Data Availability

Not applicable.
